# Long non‐coding RNA*FOXD1‐AS1* modulated CTCs epithelial‐mesenchymal transition and immune escape in hepatocellular carcinoma in vitro by sponging miR‐615‐3p

**DOI:** 10.1002/cnr2.2050

**Published:** 2024-03-22

**Authors:** Bao‐ling Guo, Qiu‐xiang Zheng, Yun‐shan Jiang, Ying Zhan, Wen‐jin Huang, Zhi‐yong Chen

**Affiliations:** ^1^ Department of Oncology Longyan First Affiliated Hospital of Fujian Medical University Longyan Fujian People's Republic of China

**Keywords:** CTC, hepatocellular carcinoma, immune escape, lncRNA *FOXD1‐AS1*, *PD‐L1*

## Abstract

**Background:**

Hepatocellular carcinoma (HCC) is widely recognized as a globally prevalent malignancy. Immunotherapy is a promising therapy for HCC patients. Increasing evidence suggests that lncRNAs are involved in HCC progression and immunotherapy.

**Aim:**

The study reveals the mechanistic role of long non‐coding RNA (lncRNA) FOXD1‐AS1 in regulating migration, invasion, circulating tumor cells (CTCs), epithelial‐mesenchymal transition (EMT), and immune escape in HCC in vitro.

**Methods:**

This study employed real‐time PCR (RT‐qPCR) to measure *FOXD1‐AS1*, miR‐615‐3p, and programmed death‐ligand 1 (*PD‐L1*). The interactions of *FOXD1‐AS1*, miR‐615‐3p, and *PD‐L1* were validated via dual‐luciferase reporter gene and ribonucleoprotein immunoprecipitation (RIP) assay. In vivo experimentation involves BALB/c mice and BALB/c nude mice to investigate the impact of HCC metastasis.

**Results:**

The upregulation of lncRNA *FOXD1‐AS1* in malignant tissues significantly correlates with poor prognosis. The investigation was implemented on the impact of lncRNA *FOXD1‐AS1* on the migratory, invasive, and EMT of HCC cells. It has been observed that the lncRNA *FOXD1‐AS1* significantly influences the generation and metastasis of ^M^CTC in vivo analysis. In mechanistic analysis, lncRNA *FOXD1‐AS1* enhanced immune escape in HCC via upregulation of *PD‐L1*, which acted as a ceRNA by sequestering miR‐615‐3p. Additionally, lncRNA *FOXD1‐AS1* was found to modulate the EMT of CTCs through the activation of the PI3K/AKT pathway.

**Conclusion:**

This study presents compelling evidence supporting the role of lncRNA *FOXD1‐AS1* as a miRNA sponge that sequesters miR‐655‐3p and protects *PD‐L1* from suppression.

## 1 INTRODUCTION

Hepatocellular Carcinoma (HCC) is widely recognized as a globally prevalent malignancy.^1^ In recent years, there has been a consistent annual rise in the incidence of HCC in China. Tumor metastasis poses a significant challenge in the clinical management of HCC.^2^ Currently, surgical intervention for HCC is predominantly restricted to the early stages of the disease. However, many HCC patients are diagnosed in the advanced stage, thus impeding the effectiveness of radiotherapy and chemotherapy as viable treatment options. The high tumor recurrence and metastasis rate are major factors contributing to the poor prognosis of HCC patients.^1^

Recent research findings have indicated that immunotherapy is a promising therapy for HCC patients.^3^ Furthermore, recent investigations have revealed that the intrinsic role of programmed death receptor 1 (*PD‐1*) is significant in developing immune resistance against tumors. In HCC cells, the activation of *PD‐1* may occur via interaction with its ligand, which appears in tumor cells. This activation regulates downstream mammalian rapamycin signaling targets, thereby accelerating cancer development, irrespective of acquired immunity. The latest research shows that the *PD‐1* signal transduction enhanced the progression of HCC cells.^4^ Despite the existing immunotherapy applications, there remains a need for in‐depth research on the regulatory function of *PD‐L1* in HCC. Polymorphisms in genes encoding the disease progression as Programmed Cell Death 1 (PD‐1) or CTLA4 or TLR9 could be candidate markers predicting treatment response warranted study.^5^, ^6^, ^7^ lncRNA is a class of RNA characterized by its length exceeding 200 nucleotides (nt) and inability to encode proteins.^8^ LncRNAs have emerged as one of the most interesting components of the transcriptome^8^ and hence, decoding HCC from a ncRNAs perspective is mandatory emerging.^9^ Increasing evidence suggests that lncRNAs are not only involved in gene expression regulations but also implicated in several biological and pathological processes, such as tumor development and its progression.^10^ New research findings have revealed that the dysregulation of lncRNA expression contributes to the developing HCC and the subsequent formation of metastasis.^11^, ^12^, ^13^, ^14^, ^15^, ^16^ It is noted that competing endogenous RNAs (ceRNAs) has been identified to mediate lncRNA activity. In this model, lncRNA or mRNA harboring the same miRNA response element can modulate each other's expression levels by competitively binding to shared miRNAs that block target mRNAs.^15^, ^16^ miRs use in the environment‐related noncommunicable diseases like HCC, nowadays for diagnosis or prognosis that could be sponged or controlled by ncRNAs as lncRNA.^17^ Nevertheless, the mechanistic role of lncRNAs regarding the progression of HCC remains poorly elucidated and necessitates further research.

This study investigates the expression of FOXD1‐AS1 in HCC. The study reveals that FOXD1‐AS1 played key role in regulating EMT and immune escape in HCC in vitro.

## 2 METHOD

### 2.1 Sample and cell lines

This research comprised 46 HCC patients who received surgical treatments during 2014 and 2017, along with adjacent normal tissues, were obtained and verified by a trained pathologist. These patients were not exposed to any treatments (chemotherapy or radiotherapy) before surgery. Informed consent was taken from all participating patients. Human normal liver cell line THLE‐3 and HCC cell lines HepG2, HepG2.2.15, Hep3B, Huh7, and Hep3B cells were purchased from the American Type Culture Collection. Dulbecco's Modified Eagle's Medium (DMEM, Gibco, United States) supplemented with 10% fetal bovine serum (FBS, Gibco) was procured for cultivating THLE‐3 and all HCC cells. The cells were maintained under the standard conditions (37°C and 5% CO_2_). Detailed clinicopathological features are described in Table 1.

**TABLE 1:** Clinicopathologic features of 46 HCC specimens.

Clinical features	*N*
Sex	
Female	12
Male	34
Age	
>55	20
≤55	26
Vascular invasion	
Present	21
Absent	25
Distant metastasis	
Present	28
Absent	18
Lymph node metastasis	
Present	16
Absent	30
TNM stage	
I‐II	16
I‐I‐IV	30
Cirrhosis	
Yes	19
No	27
Treatment after the surgery	
TACE	39
Radiotherapy	12
Chemotherapy	0
Immune therapy	0
Target therapy	0

Abbreviation: HCC, hepatocellular carcinoma; TACE, Transcatheter arterial chemoembolization.

### 2.2 Cell transfection

Gene Chem (Shanghai, China) provided all experiment interference vectors. The transfections were conducted utilizing Lipofectamine 2000 (Sigma) into 5 × 10^6^ HCC cells. The transfection efficiency was evaluated by harvesting cells after 48 h transfection. All these procedures adhered to the manufacturer's guidelines.

### 2.3 Cell proliferation

A total of 4 × 10^4^ indicated treated HCC cells in 100 μL cell suspension were added into each well of 96‐well plates. The cell viability was analyzed via Cell Counting Kit (Yeasen, China) (CCK)‐8 assay. The absorbance was taken at 450 nm. The execution of all procedural stages adheres strictly to the guidelines outlined in the manual provided within the kit. The operational procedures strictly adhered to the guidelines outlined in the manual.

### 2.4 Transwell assay

Cell invasion was examined by adding a dilution of serum‐free medium and Matrigel (BD Biosciences, United States) to the upper chamber of a Transwell plate. The plate was subsequently placed at 37°C overnight. Matrigel was intentionally removed during the cell migration analysis. The cell suspension, containing 3 × 10^4^ indicated HCC cells, was introduced into the upper chamber. Simultaneously, 500 μL culture DMEM was introduced into the lower chamber. After 24 h incubation, the medium comprising migrated or invasive cells were removed from the lower chamber, and the cells were visualized using an inverted microscope (Nikon, Japan).

### 2.5 RIP assay

Magna ribonucleoprotein immunoprecipitation (RIP) RNA‐Binding Protein Immunoprecipitation Kit (#17–701, Millipore, Bedford, MA, United States) was used to conduct the RIP assay, following the established protocol.^18^ In brief, the magnetic beads and anti‐Ago2 antibody (Abcam) were added into cells and incubated for 24 h. Then, the proteinase K and the phenol‐chloroform‐isoamyl alcohol reagent were added for purifying RNAs.

### 2.6 Luciferase reporter assay

The *FOXD1‐AS1* fragments containing wild‐type (WT) and mutant (MUT) miR‐570‐3p binding pockets were identified and developed by Shanghai GenePharma. Subsequently, luciferase reporter assays were conducted following established protocols.^18^

### 2.7 RT‐qPCR analysis

TRIzol (Ambion, United States) was utilized to extract total RNA. The SYBR Green qPCR Master Mix (MedChem Express, NJ, United States) was utilized for all RT‐qPCR analysis. The 7900HT Fast Real‐Time PCR (Thermo Fisher Scientific, MA, United States) was performed to evaluate amplification reactions. As a control, GAPDH and U6 were utilized for normalization. Relative gene expression was quantified using 2^−ΔΔCT^ method. All premiers were shown as shown in Table 2.

**TABLE 2:** Primer sequences used for real‐time PCR.

Gene	Sequence
FOXD1‐AS1‐F	TTTTAACGCCTGGACCTGAGAAT
FOXD1‐AS1‐R	GTTAATAACGCTATGCTACAGCC
U6‐F	CTCGCTTCGGCAGCACA
U6‐R	AACGCTTCACGAATTTGCGT
E‐cadherin‐F	TGCTCTTCCAGGAACCTCTGT
E‐cadherin‐R	GTAAGCGATGGCGGCATTGTA
N‐cadherin‐F	GCGTCTGTAGAGGCTTCTGG
N‐cadherin‐R	GCCACTTGCCACTTTTCCTG
Vimentin‐F	AGGCAAAGCAGGAGTCCACTGA
Vimentin‐R	ATCTGGCGTTCCAGGGACTCAT
PD‐L1‐F	TGGCATTTGCTGAACGCATTT
PD‐L1‐R	TGCAGCCAGGTCTAATTGTTTT
PRF1‐F	GGCTGGACGTGACTCCTAAG
PRF1‐R	CTGGGTGGAGGCGTTGAAG
GZMB‐F	CCCTGGGAAAACACTCACACA
GZMB‐R	GCACAACTCAATGGTACTGTCG
GNLY‐F	CAGGCTCCCTGCCCATAAAA
GNLY‐R	CTCAAGGCCTGGGTTGCC
IFN‐γ‐F	TCGGTAACTGACTTGAATGTCCA
IFN‐γ‐R	TCGCTTCCCTGTTTTAGCTGC
miR‐655‐3p‐F	CAATCCTTACTCCAGCCAC
miR‐655‐3p‐R	GTGTCTTAAGGCTAGGCCTA
GAPDH‐F	GAGTCAACGGATTTGGTCGT
GAPDH‐R	TTGATTTTGGAGGGATCTCG

### 2.8 ELISA

The Human TNF‐α Quantikine ELISA kit and Human Serpin INF‐γ Quantikine ELISA kit awere purchased from R&D Systems Inc., to detect the concentrations of TNF‐α and INF‐γ in the CM, according to the manufacturer's instructions. The absorbance at 450 nm was measured using a microplate reader and 540 nm was set as the reference wavelength.

### 2.9 HPBMC isolation and activation

Human peripheral blood mononuclear cells (HPBMCs) were obtained from healthy participants at the same institute as mentioned above. Based on the manufacturer's instructions, HPBMCs were harvested via Lymphoprep (Stemcell Technologies, Vancouver, Canada) as previously described.^19^ Briefly, the diluted blood was carefully added on the top of Lymphoprep. After centrifuging, the mononuclear cell layer at the plasma: Lymphoprep interface was isolated and washed once with Hanks' Balanced Salt Solution. Typically, 1 × 10^6^ HPBMC was activated with 25 mL ImmunoCult Human CD3/CD28 T Cell Activator (STEMCELL, catalog no. 10971) in 1 mL ImmunoCult‐XF T Cell Expansion Medium (STEMCELL, catalog no. 10981) supplemented with 10 ng/mL IL2 (Peprotech, catalog no. 200‐02).

### 2.10 In vivo metastasis assay

In vivo metastatic ability of indicated cells was determined by tail vein injection of the cells into 4‐week‐old female nude mice (*N* = 5 for each group, mice weight = 19.93 ± 0.28 g) as previously described.^20^ Eight weeks after injection, mice were sacrificed and examined for lung metastasis using standard histological examination.

### 2.11 Hematoxylin and eosin

Incubate the slides with hematoxylin solution in a staining jar for 10 min at room temperature. Transfer the slides to a staining jar with running water (tap water is fine) till the water is clear. Transfer the slides to a staining jar with eosin solution for 3 min at room temperature. Then transfer the slides into staining jars with 70% ethanol for 20 s, 90% ethanol for 20 s, 100% ethanol for 1 min, and xylene for 3 min. Take out slides from xylene and place the slides in a fume hood till the slides are dry. Store the slides at room temperature.

### 2.12 Western blot

Proteins were extracted from a specific extraction reagent (Thermo Fisher Scientific). A fraction of these proteins were separated and subsequently transferred onto Immobilon TM‐P membranes (Merck Millipore, Billerica, United States). Rabbit‐specific PD‐L1, p‐AKT, AKT, p‐PI3K, PI3K, and Actin (Abcam) primary antibodies were placed for incubation at 4°C overnight. Membranes were probed with horseradish peroxidase (HRP)‐linked goat anti‐rabbit IgG (1: 2000, Abcam) for 1 h after washing with TBST. The antibody interaction was detected by enhanced chemiluminescence.

### 2.13 CTCs isolation and identification

The isolation and enrichment of circulating tumor cells (CTCs) were performed by a CTCBIOPSY device (Wuhan YZY Medical Science and Technology Co., Ltd., Wuhan, China). According to the manufacturer's instructions, we diluted 1 mL mouse blood into 5 mL of 0.9% sodium chloride solution, and the total liquid was then transferred to ISET tubes with an 8 μm diameter aperture membrane. Through positive pressure from 12 to 20 mmHg in ISET tubes, candidate CTCs were adhered to the ISET tube membrane and identified by three‐color immunofluorescence.

### 2.14 PI3K activity assay

PI3K kinase activity assay was performed as the kit protocol described previously.^21^

### 2.15 Statistical analysis

Data was statistically examined via GraphPad Prism 6.0 software (GraphPad Software, United States) as mean ± standard deviation. Statistical tests including unpaired t‐test and one‐way ANOVA were utilized to measure differences between two groups, or multiple groups respectively. Survival curves were calculated according to the Kaplan–Meier method and were compared by a log‐rank test. A threshold of *p* < .05 was considered for determining the significance level.

## 3 RESULTS

### 3.1 Upregulation of LncRNA *FOXD1‐AS1* in HCC

**FIGURE 1:**
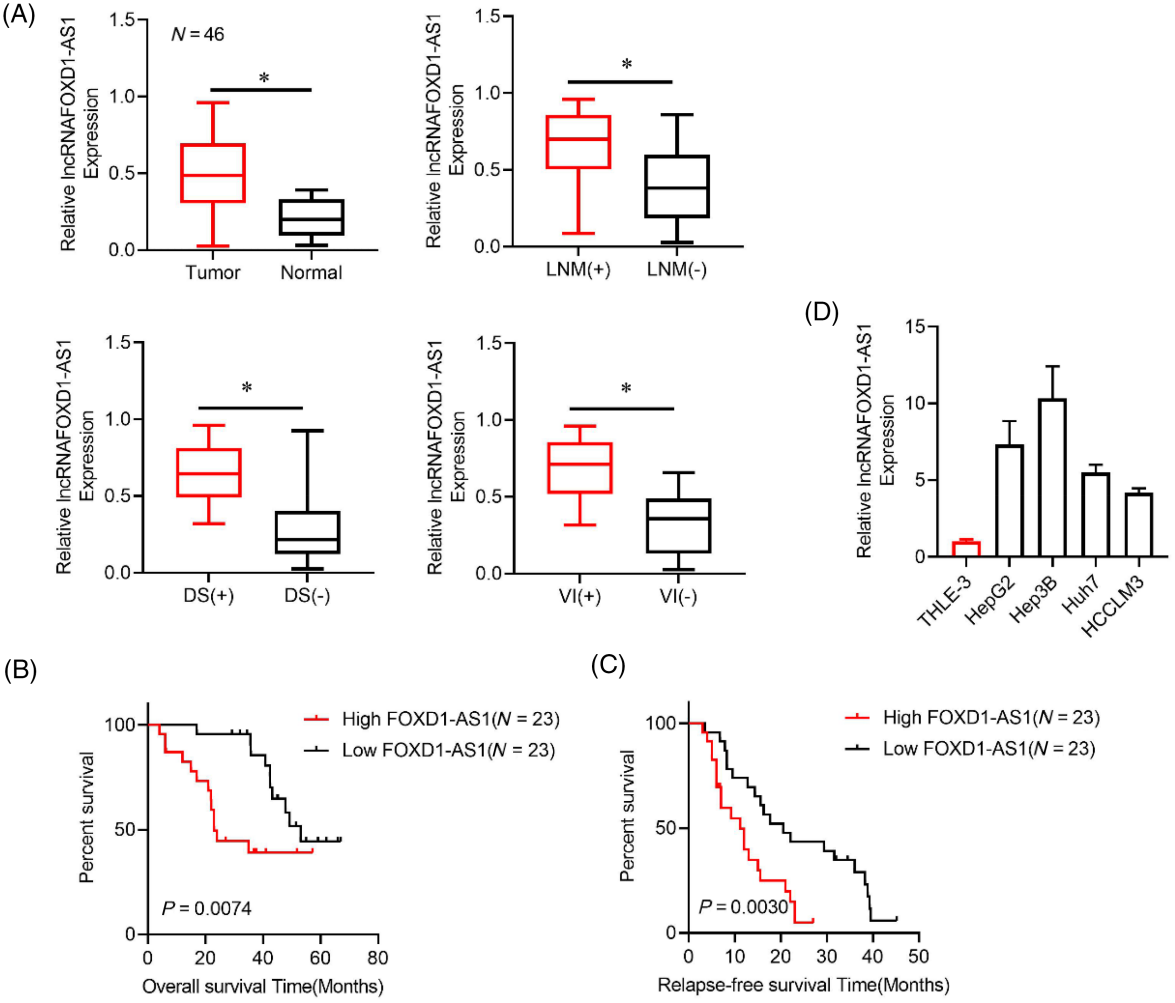
Upregulation of lncRNA *FOXD1‐AS1* in HCC. (A) Analysis of LncRNA *FOXD1‐AS1* in HCC tissues via qPolymerase Chain Reaction (PCR). (B, C) Kaplan–Meier examination of the Overall survival (OS) and Recurence free survival (RFS) of 46 HCC patients. (D) Analysis of LncRNA *FOXD1‐AS1* in HCC cell lines via qPCR. Representative data were derived from three distinct experiments with *p < .05, SD, and error bars. For (A) student *t*‐test; (B, C) log‐rank test. DS, distant metastasis; HCC, hepatocellular carcinoma; LNM, lymph node metastasis.

The findings revealed a substantial rise in the level of *FOXD1‐AS1* in tumor tissues compared to adjacent tissues (Figure 1A). Subsequent results showed a strong link between the level of *FOXD1‐AS1* and lymph nodes, distant metastasis, or vascular penetration (Figure 1A). In addition, the findings demonstrated a substantial association between elevated levels of *FOXD1‐AS1* and adverse prognostic outcomes (Figure 1B,C). Consequently, four HCC cell lines validated the upregulated levels of *FOXD1‐AS1* in all HCC cell lines (Figure 1D). The findings indicate a positive connection between the overexpression of *FOXD1‐AS1* and adverse clinical prognoses among HCC patients.

### 3.2 Role of lncRNA *FOXD1* on the migratory, invasive, and EMT properties of HCC cells

**FIGURE 2:**
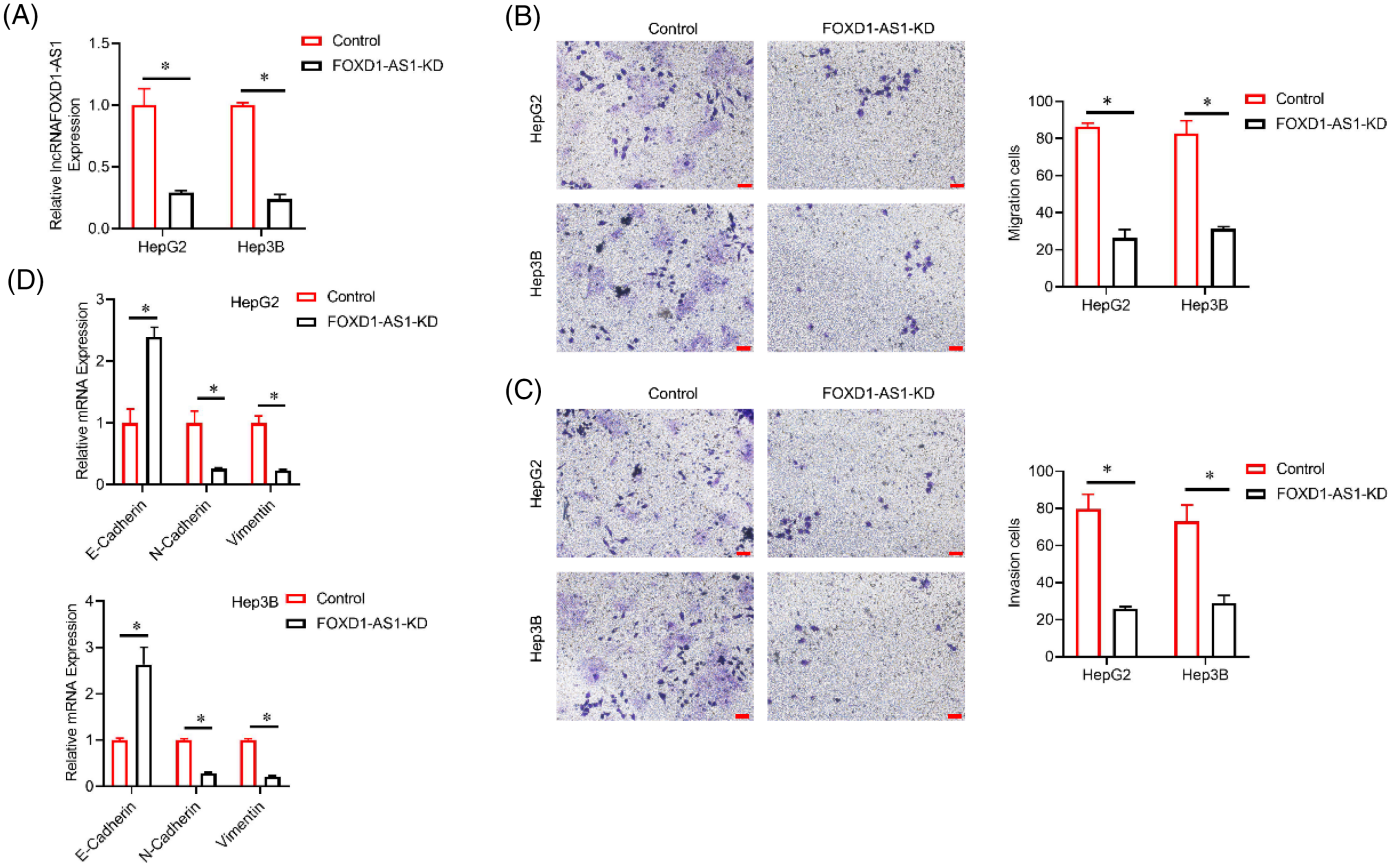
Role of *FOXD1‐AS1* on the migratory, invasive, and EMT properties of HCC cells. (A) lncRNA *FOXD1‐AS1* level in all treated groups. (B, C) Evaluation of the migratory and invasive abilities of CRC cells via Transwell assay following various treatments. Bar scale = 50 μM. (D) EMT corresponding gene and protein levels. Representative analyses were derived from three distinct experiments with *p < .05, SD, and error bars. For (A–D) student *t*‐test. EMT, epithelial‐mesenchymal transition; HCC, hepatocellular carcinoma.

Loss function approaches were applied to elucidate the role of *FOXD1‐AS1* on the progression of tumors. First, qRT‐PCR was executed to ascertain the higher efficacy of the optimal *FOXD1‐AS1* HCC cell line (Figure 2A). Transwell migration and invasion assays revealed the inhibitory effect of *FOXD1‐AS1* knockdown on the migration and invasion of HCC cells (Figure 2B,C). In light of the substantial role of EMT in HCC cell migration and invasion, this study investigated to determine whether *FOXD1‐AS1* can induce EMT in HCC cells.^22^ The findings displayed that *FOXD1* knockdown decreased the vimentin and N‐cadherin levels and enhanced E‐cadherin levels in HCC cells (Figure 2D). These results collectively indicate that *FOXD1‐AS1* can regulate HCC cell migration and invasion by modulating the EMT process.

### 3.3 *FOXD1‐AS1* facilitates the immune escape of HCC via PDL‐1

**FIGURE 3:**
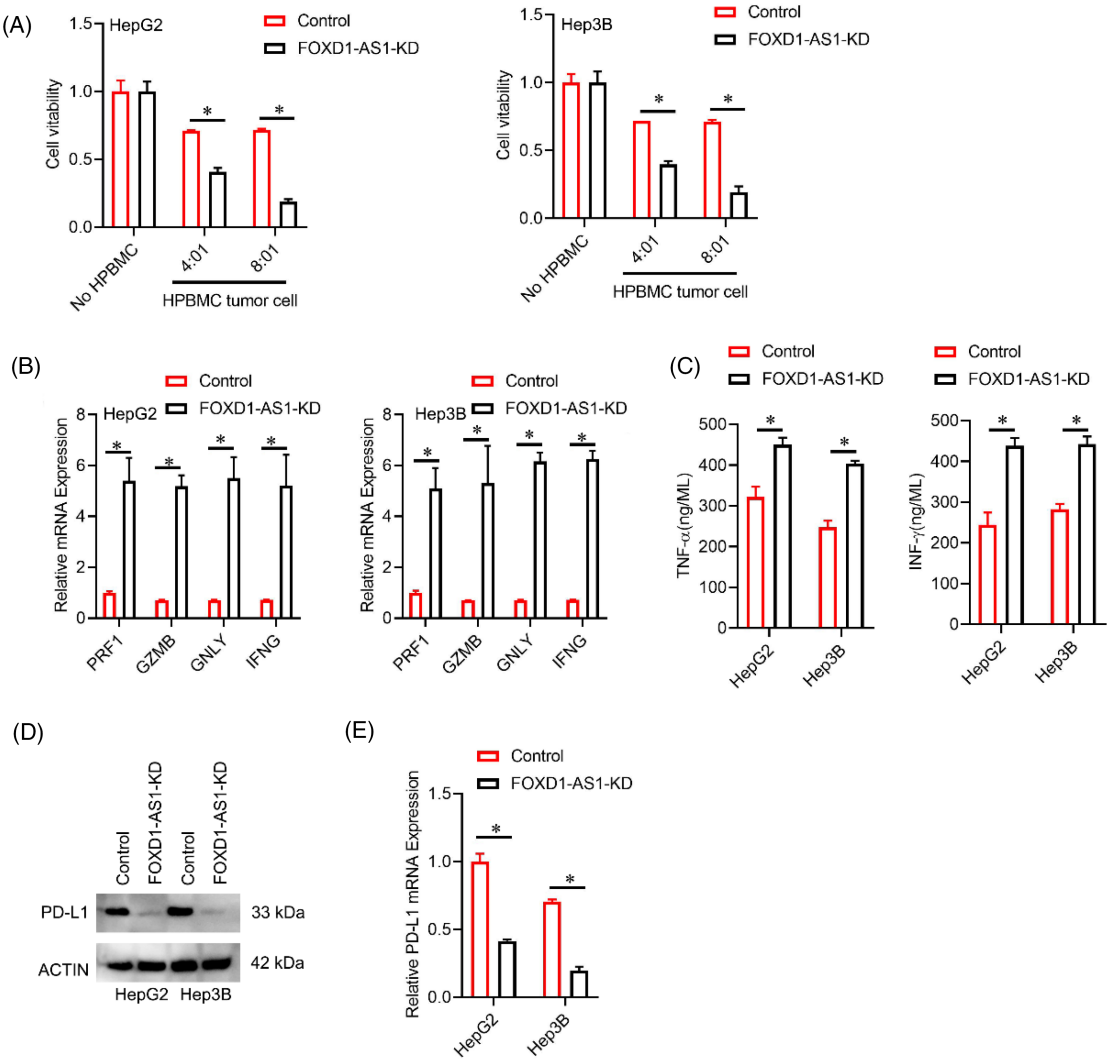
*FOXD1‐AS1* facilitates immune escape of HCC via *PDL‐1*. (A) CCK8 kit analysis for tumor cell elimination by HPBMC. Co‐culturing of HCC cells with and without HPBMC. Data normalization with their respective controls. (B) Detection of *PRF1, GZMB, GNLY*, and *IFN‐γ* via RT‐PCR in HPBMC co‐cultured with HCC cells with control or *FOXD1‐AS1‐*KD. (C) The TNF‐α and IFN‐*γ* concentration in culture medium from different transfected groups was determined by ELISA. (D, E) Levels of protein and mRNA of *PD‐L1* in HCC cells. Representative analyses were derived from three distinct experiments with *p < .05, SD, and error bars. For (A–C) and (E) student *t*‐test. HCC, Hepatocellular carcinoma; HPBMC, human peripheral blood mononuclear cells.

Based on other research findings, alterations within the tumor microenvironment (TME) hold significant implications for tumor pathogenesis. To validate the function of *FOXD1‐AS1* in HCC cells, HCC cells were co‐cultured with activated T cells. Besides, a considerable enhancement of T cell‐mediated cell killing was observed upon knockdown of *FOXD1‐AS1* (Figure 3A). Additionally, the levels of mRNA for *PRF1* (perforin 1), *GZMB* (granzyme B), *GNLY* (granulysin), and *IFN‐γ* in PBMCs exhibited upregulation following co‐culturing with cells upon *FOXD1‐AS1* knockdown (Figure 3B). ELISA assay indicated that secretion level *TNF‐a* and *INF‐γ* was also increased in PBMCs co‐culturing with cells upon *FOXD1‐AS1* knockdown (Figure 3C). The co‐expression of *PD‐L1* on tumor cells and *PD‐1* on T lymphocytes leads to the inhibition of T cell activity, consequently evading immune surveillance.^23^ Henceforth, this research explored the involvement of dysregulated *PD‐L1* expression in *FOXD1‐AS1*‐mediated tumor development. The *PD‐L1* expressions were examined in each treatment group that was co‐cultured with T cells. The findings revealed a substantial protein reduction of *PD‐L1* in the treated group while the *FOXD1‐AS1* expression was knocked down (Figure 3D). Additionally, the mRNA levels of *PD‐L1* were also significantly reduced in this treatment group (Figure 3E).

### 3.4 *FOXD1‐AS1* improved *PD‐L1* expression by sponging miR‐615‐3p as a ceRNA in vitro

**FIGURE 4:**
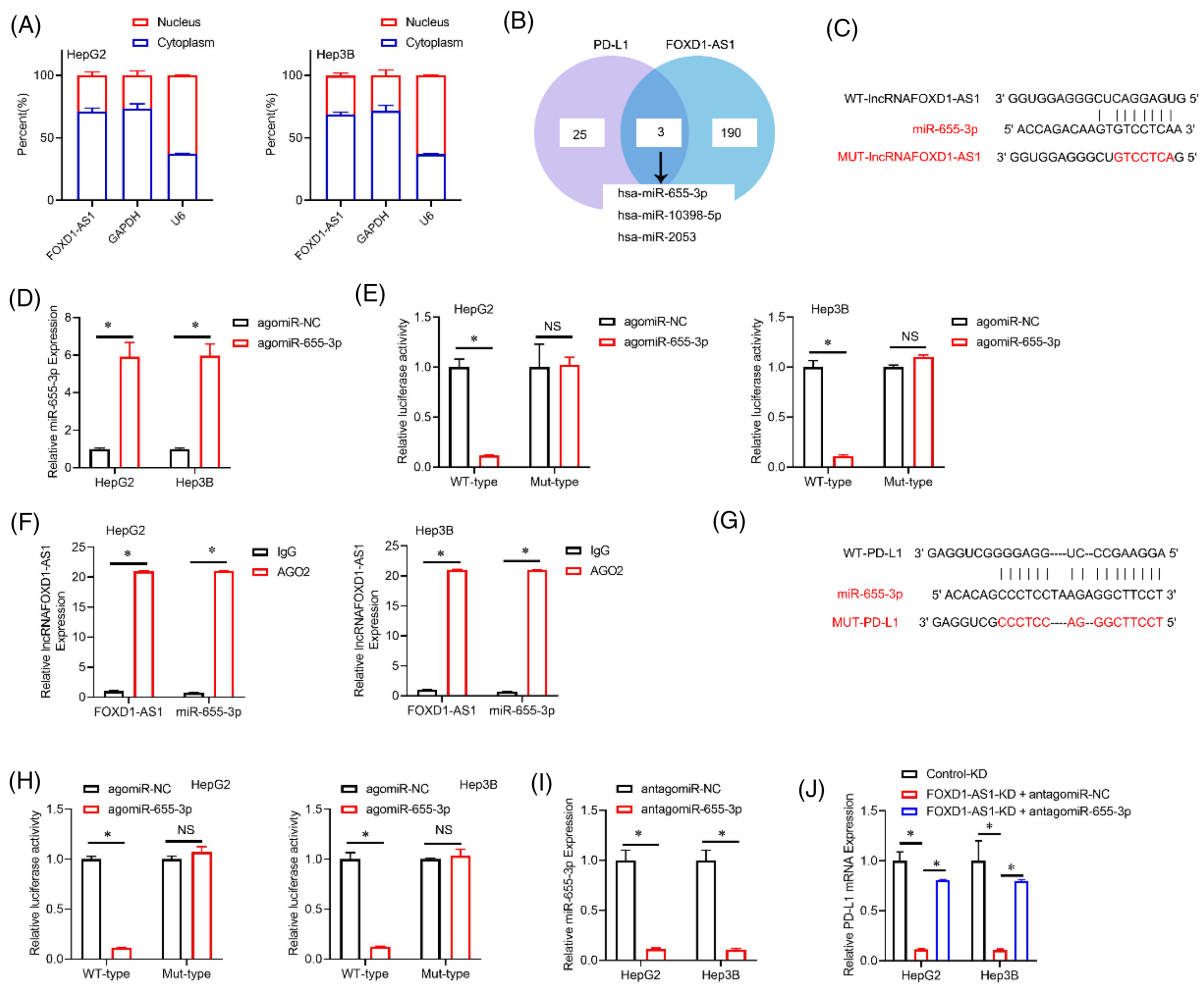
*FOXD1‐AS1* improved *PD‐L1* expression by sponging miR‐615‐3p as a ceRNA in vitro. (A) Localization of *FOXD1‐AS1* in the cytoplasm of HCC cells. (B) Binding interaction of miR‐615‐3p with *FOXD1‐AS1* and *PD‐L1* via Venn diagram. (C) Identification of miR‐615‐3p binding sites within *FOXD1‐AS1* via bioinformatics analysis. (D) Expression of miR‐615‐3p via RT‐qPCR. (E) Respective HCC cell lines with luciferase reporter assays. (F) Enrichment of *FOXD1‐AS1* and miR‐615‐3p in Ago2‐immunoprecipitates in contrast to IgG control. (G) Identification of miR‐615‐3p binding sites within *PD‐1* via bioinformatics analysis. (H) Respective HCC cell lines with luciferase reporter assays. (I) Detection of miR‐615‐3p in HCC cells transfected with antagomiR‐615‐3p or antagomir‐NC via RT‐qPCR. (J) Measurement of *PD‐L1* mRNA levels via RT‐qPCR. β‐actin was used to normalize the data as △^Ct^ and examined by Spearman's correlation analysis. Representative analyses were derived *N* = 3 from three distinct experiments with *p < .05, SD, and error bars. For (D–F), (H, I) student *t*‐test. For (J) one‐way ANOVA test. HCC, hepatocellular carcinoma.

Examination of subcellular fractionation revealed predominant cytoplasmic localization of lncRNA *FOXD1‐AS1* in HCC cells (Figure 4A). Based on this observation, lncRNA *FOXD1‐AS1* functions as a ceRNA for a miRNA that regulates the expression of *PD‐L1*. Various miRNAs that share complementary binding sites with the *FOXD1‐AS1* and *PD‐L1*, were uncovered (miR‐615‐3p, miR‐10398‐5p, and miR‐2053) as potential candidates (Figure 4B). To conduct a more comprehensive investigation, agomiR‐NC, agomiR‐615‐3p, agomiR‐10 398‐5p, or agomiR‐2053 were con‐transfected in combination with MUT‐lncRNA *FOXD1‐AS1* or WT‐lncRNA *FOXD1‐AS1* variants in HCC cells. These transfections were carried out to validate the effects of luciferase reporter assays. As depicted in Figure 4C–E, agomiR‐615‐3p resulted in an inhibition of luciferase activity specifically in HCC cells. This reduction was noted in the WT‐lncRNA *FOXD1‐AS1* construct, while no substantial impact was observed on the activity of the MUT‐lncRNA *FOXD1‐AS1* construct. However, it was also noted that the agomiR‐10 398‐5p or agomiR‐2053 failed to result in a decrease in luciferase activity when examining the WT‐lncRNA *FOXD1‐AS1* or MUT‐lncRNA *FOXD1‐AS1* construct in PC cells (Figure S1A–F). Furthermore, the *FOXD1‐AS1* and miR 615‐3p exhibited considerable enrichment in Ago2‐immunoprecipitates in comparison to the control, as evidenced by the results from the RIP assays (Figure 4F). To confirm that *PD‐L1* is a target of miR‐615‐3p, the full‐length 3′‐untranslated regions (3′‐UTR) of the *PD‐L1* gene were inserted into the downstream of the Renilla luciferase gene (Figure 4G). Consistently, the use of agomiR‐615‐3p resulted in a decreased luciferase activity in HCC cells of the WT‐PD‐L1 construct. However, no significant effect was noted on the MUT‐PD‐L1 construct (Figure 4H). As depicted in Figure 4I,J, the use of antagomiR‐615‐3p in HCC cells with suppressed *FOXD1‐AS1* resulted in the restoration of decreased levels of PD‐L1 mRNA. This observation suggests that *FOXD1‐AS1* promotes the *PD‐L1* expression by sequestering miR‐615‐3p as a ceRNA.

### 3.5 *FOXD1‐AS1* modulated EMT via PI3K/AKT pathway

**FIGURE 5:**
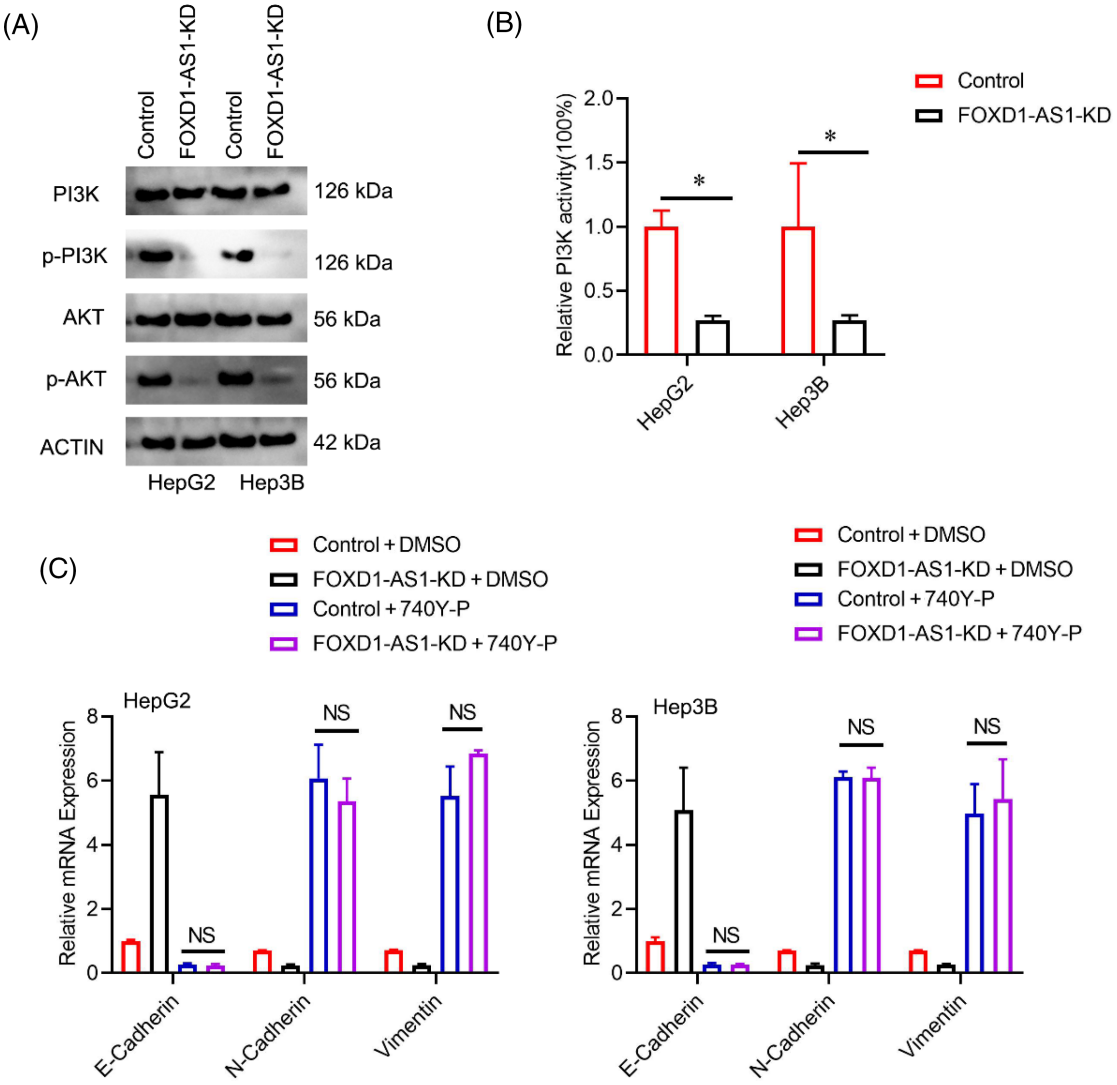
lncRNA *FOXD1‐AS1* regulated EMT via PI3K/AKT pathway. (A) Respective level of *PI3K, AKT, p‐PI3K*, and *p‐AKT* expressions in corresponding HCC cells. (B) PI3K kinase activity evaluation in the desired cell. (C) Respective level of E‐cadherin, N‐cadherin, and vimentin in corresponding HCC cells with or without treatment of 740Y‐P. Representative analyses were derived from three distinct experiments with *p < .05; NS *p* > .05, SD, and error bars. For (B, C) student *t*‐test. EMT, epithelial‐mesenchymal transition; HCC, hepatocellular carcinoma.

Existing scientific inquiries have consistently suggested a strong association between the PI3K/AKT signaling pathway and EMT. Thus, this study investigated whether *FOXD1‐AS1* exerts its influence on EMT by modulating the PI3K/AKT pathway. The impact of *FOXD1‐AS1* knockdown on the protein levels of phospho‐AKT demonstrated a remarkable reduction in the *FOXD1‐AS1* knockdown cells (Figure 5A). In addition, the assays related to kinase activity provided evidence suggesting that the PI3K regulation was influenced by *FOXD1‐AS1* (Figure 5B). To investigate the PI3K/AKT pathway activation in facilitating the stimulation of EMT by *FOXD1‐AS1*, HCC cells with suppressed expression of *FOXD1‐AS1* were noted after treatment with 20 uM of PI3K/AKT pathway activator 740Y‐P. The outcomes demonstrated that the addition of 740Y‐P *in FOXD1‐AS1*‐knockdown cells resulted in the restoration of E‐cadherin, N‐cadherin, vimentin, and snail expression (Figure 5C). These results suggest that lncRNA *FOXD1‐AS1* contributes to the regulation of EMT via the PI3K/AKT pathway.

### 3.6 *FOXD1‐AS1* affects in vivo ^M^CTC generation and metastasis

**FIGURE 6:**
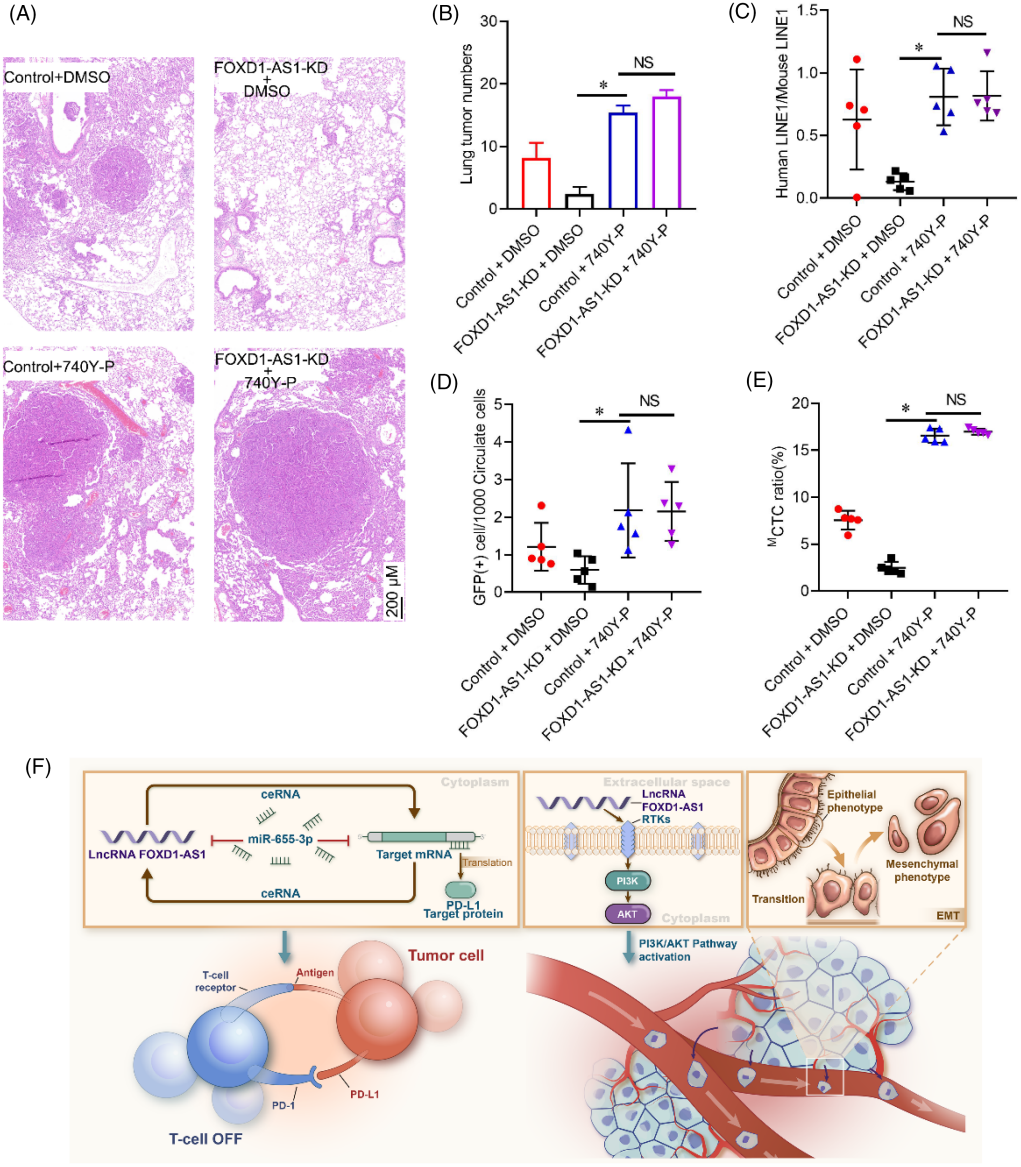
*FOXD1‐AS1* affects in vivo ^M^CTC generation and metastasis. (A) Representative H&E stained images of lung metastatic loci from each group. Bar scale = 200 μM. (B) Quantitative analysis pulmonary metastatic foci in indicated mice group. (C) The relative levels of human LINE1 DNA were analyzed by qPCR of genomic DNA from whole‐blood samples 48 days after orthotopic xenograft transplantation of the indicated group; expression levels were normalized to mouse LINE1 DNA. (D) The number of GFP‐labeled CTCs was examined from whole‐blood samples. (E) Evaluation of ^M^CTC ratio in the blood sample of different groups. (F) Schematic diagram illustrated the role of *FOXD1‐AS1* modulated CTCs of EMT and immune escape in HCC. Representative analyses were derived from three distinct experiments with *p < .05; NS *p* > .05, SD, and error bars. For (B–E) one‐way ANOVA test. CTCs, circulating tumor cells; EMT, epithelial‐mesenchymal transition; HCC, hepatocellular carcinoma; H&E, hematoxylin and eosin.

To validate the above in vivo results, stable cell lines including control‐KD + DMSO, *FOXD1‐AS1*‐KD + DMSO, control‐KD + 740Y‐P, and *FOXD1‐AS1*‐KD + 740Y‐P were individually injected via tail vein of mice to investigate the effect of *FOXD1* on metastasis. *FOXD1* knockdown significantly suppressed HCC metastatic, and this suppressive impact of *FOXD1* was counteracted by the use of 740Y‐P (Figure 6A,B). We further evaluated human LINE1 DNA, an indicator of CTCs, in the whole‐blood samples and calculated its ratio to mouse LINE1 DNA to reflect the number of CTCs. As shown in Figure 6C, mice transplanted with *FOXD1* knockdown significantly showed less human LINE1 DNA and this suppressive impact of *FOXD1* was counteracted by the use of 740Y‐P. We further used flow cytometry to examine the numbers of CTCs from whole‐blood samples obtained from the orthotopic model, and *FOXD1* knockdown significantly decreased the number of CTCs and this suppressive impact of *FOXD1* was counteracted by the use of 740Y‐P (Figure 6D). The process of EMT facilitates the removal of cancer cells from primary tumors and their passage into the bloodstream, leading to the formation of CTCs and subsequent metastatic tumor growth. *FOXD1* knockdown resulted in a notable reduction in the rate of ^M^CTC generation when compared to the negative control. Treatment with 740Y‐P was able to rescue the inhibitory effect of *FOXD1* knockdown on ^M^CTC generation (Figure 6E). These findings suggest that the expression of *FOXD1* is involved in influencing ^M^CTC generation and metastasis in vivo.

## 4 DISCUSSION

This study illustrates a novel immune escape mechanism in HCC, in which lncRNA *FOXD1‐AS1* modulates immune escape. The findings demonstrate that lncRNA *FOXD1‐AS1* is involved in tumor growth and acts as a ceRNA by sequestering miR‐615‐3p, leading to increased *PD‐L1* levels. This, in turn, impairs the CD8+ T‐cell response, ultimately facilitating the progression of HCC. This study aims to establish a comprehensive reference basis for upcoming advancements in tumor immunotherapy and targeted therapy.

lncRNA was first regarded as a by‐product of the transcription mediated by RNA polymerase II. It was considered to be a form of “noise” without biological function. Current investigations have revealed that lncRNA can modulate gene expression via various mechanisms, including epigenetic regulation, transcriptional regulation, and posttranscriptional regulation. These outcomes highlight the involvement of lncRNA in the pathological progression of numerous diseases, particularly malignant tumors.^8^, ^24^, ^25^, ^26^, ^27^ Moreover, lncRNAs exhibit the function of ceRNA, engaging in intricate interactions with miRNAs and modulating mRNA expression to exert significant biological roles. The present research has unveiled that the lncRNA *FOXD1‐AS1* exhibits an elevated expression in HCC tissues, particularly in aggressive cases. Furthermore, the overexpression of lncRNA *FOXD1‐AS1* is significantly linked with adverse clinical prognosis in patients with HCC.

TME is a complex composition of diverse cellular populations, extracellular matrix (ECM), growth factors, proteolytic enzymes, and their inhibitors, and signaling molecules.^28^ TME significantly affects tumor development, metastasis, and diagnosis primarily through its interactions with cancerous cells. The regulation of the origin, development, penetration, and metastasis of TME is influenced by many factors, including ECM, growth factors, and immune cells in the microenvironment. The impact of TME on tumor progression, metastasis, and prognosis is widely known, primarily via its influence on the cellular immune microenvironment.^29^ Tumor cells can evade the immune system and develop immune tolerance via diverse mechanisms, such as exosome secretions.^30^ Several investigations have examined the intricate interaction between the TME and CD8+ T cells.^31^ Multiple investigations have demonstrated that *PD‐1* is the primary inhibitory epitope for tumor immunity. Immunotherapeutic interventions targeting *PD‐1* and its ligand, *PD‐L1*, have emerged as a potent therapeutic approach for tumor management.^32^ This study presents the first report and validates the regulatory effect of lncRNA *FOXD1‐AS1* in enhancing the *PD‐L1* expression by sequestering miR‐615‐3p as a ceRNA and inhibiting CD8+ T‐cell response, indicating that lncRNA FOXD1‐AS1 might play import role in immune escape in HCC in vitro.

Metastasis, an intricate process, comprises a series of multiple steps induced by various mechanisms.^33^, ^34^, ^35^, ^36^ Extensive evidence has supported that CTCs are the precursors to metastatic lesions.^37^ The EMT process is involved in facilitating the escape of CTCs and granting them several pro‐metastatic traits^38^ and is intricately involved in the entire cascade of metastasis.^39^ In general, EMT has been noticed to have a role in the initial dissemination of CTCs during metastasis.^33^ In addition, epithelial‐like CTCs (ECTCs) can acquire mesenchymal characteristics via EMT. This phenotypic transition enhances their invasive capacity, thus overcoming barriers that prevent metastasis^34^ and exhibit great potential for developing metastatic lesions.^35^ During the process of EMT, there is a notable reduction of *E‐cadherin* along with upregulation of *N‐cadherin* and *Vimentin*.^36^ Growing evidence has demonstrated a positive correlation between the CTCs numbers, specifically mesenchymal‐like CTCs or ^M^CTCs, and the development of tumor metastasis.^37^ Further elucidation of EMT is essential to enhance in‐depth knowledge of the mechanisms regarding liver metastasis in HCC. This will provide valuable insights into the alteration of CTCs and facilitate an in‐depth understanding of HCC metastasis. This research illustrates that lncRNAFOXD1‐AS1 influences the EMT, ^M^CTC generation, and metastasis via modulation of the PI3K/AKT pathway. The current study was intended to expand the cognitive understanding of the epigenetic regulation‐mediated EMT mechanism.

Despite extensive in vivo and in vitro validation, this research currently lacks the clinical information that provides corresponding support. Therefore, it is necessary to implement clinical sample identification and interventions to improve the treatment of HCC and enhance the overall surveillance of patients. Meanwhile, it will be to use an immunocompetent HCC model such as C57BL/6 mice with orthotopic Hepa1‐6 HCC model or humanized mice HCC model to explore FOXD1‐AS1 involved in immune escape in HCC.

## 5 CONCLUSION

In conclusion, evidence supports the role of lncRNA *FOXD1‐AS1* as a microRNA sponge, sequestering miR‐655‐3p on Ago2, thus preventing *PD‐L1* from suppression and influencing immune escape in HCC in vitro. Also, lncRNA *FOXD1‐AS1* regulates EMT through the PI3K/AKT pathway and enhances HCC migration, invasion, ^M^CTC generation, and metastasis. This novel approach offers an opportunity to investigate the role of immune escape in HCC (Figure 6F). Consider vitamin E as prophylactic better than D for inflammation and oxidative stress as previous reported,^40^ the future prospective and recommendation was whether vitamin E play a role in the treatment of HCC by regulating the lncRNA FOXD1‐AS1 expression.

## AUTHOR CONTRIBUTIONS

**Bao‐ling guo:** Conceptualization; investigation; writing – review and editing; writing – original draft. **Qiu‐xiang Zheng:** Writing – review and editing; writing – original draft; conceptualization; methodology. **Yun‐shan Jiang:** Conceptualization; methodology; writing – review and editing; writing – original draft. **Ying Zhan:** Conceptualization; data curation. **Wen‐jin Huang:** Writing – original draft; writing – review and editing; methodology; formal analysis. **Zhi‐yong Chen:** Conceptualization; methodology; data curation; investigation; funding acquisition; writing – review and editing; writing – original draft; resources.

## FUNDING INFORMATION

This study was supported by Longyan City Science and Technology Plan Project (2020LYF17043).

## CONFLICT OF INTEREST STATEMENT

The authors declare that they have no conflict of interest.

## ETHICS STATEMENT

The authors are accountable for all aspects of the work and for ensuring that questions related to the accuracy or integrity of any part of the work are appropriately investigated and resolved. The study was conducted in accordance with the Declaration of Helsinki (as revised in 2013). The Ethics Committee of Longyan First Affiliated Hospital of Fujian Medical University (No. 2020LYF17043, Longyan, Fujian, China) approved this collection procedure and participants gave informed consent. All methods were carried out in accordance with relevant guidelines and regulations for human studies. Animal studies were conducted under standards for the National Institutes of Health guide for the care and use of Laboratory animals (NIH Publications No. 8023, revised 1978), and the Longyan First Affiliated Hospital of Fujian Medical University Ethics Committee (No. 2020LYF17043, Longyan, Fujian, China) approved the methods. The maximal tumor size was less than 2000 mm^3^ permitted the Animal Care and Use Ethics Committee of Longyan First Affiliated Hospital of Fujian Medical University (Longyan, Fujian, China). The maximal tumor size in all mice involved in this study was not exceeded than 2000 mm^3^.

## INFORMED CONSENT

All patients declared their participation and written consent was obtained before participation in the study.

## Supporting information


**Data S1.** Supporting Information.

## Data Availability

The data that support the findings of this study are available from the corresponding author upon reasonable request.
