# Supplementary material for: Long non‐coding RNAFOXD1‐AS1 modulated CTCs epithelial‐mesenchymal transition and immune escape in hepatocellular carcinoma in vitro by sponging miR‐615‐3p
Source: Cancer Rep (Hoboken). 2024 Mar 22;7(3):e2050. doi: 10.1002/cnr2.2050 (PMC10959247; doi:10.1002/cnr2.2050)
Supplement: Supplementary file 1 — Data S1. Supporting Information. [file CNR2-7-e2050-s001.docx]

**Supplement information**

**Long non-coding RNA*FOXD1-AS1* modulated CTCs epithelial-mesenchymal transition and** **Immune Escape in Hepatocellular Carcinoma *in vitro* by** **sponging miR-615-3p**

**
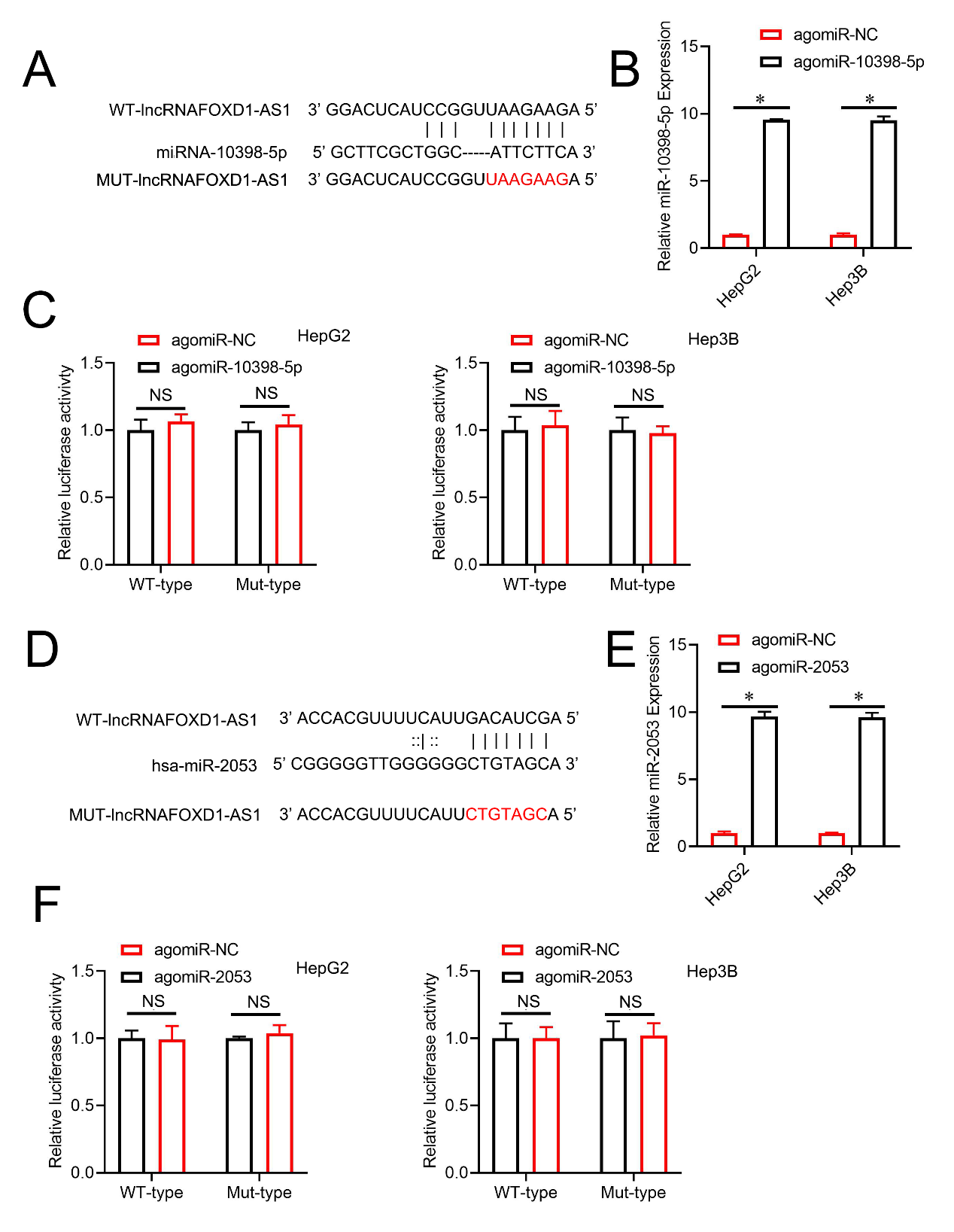
Figure S1** (A) Binding interaction between miR-10398-5p and lncRNA *FOXD1-AS1 via* bioinformatics analysis. (B) Expression of miR-10398-5p *via* RT-qPCR analysis. (C) PC cell lines detection by Luciferase reporter assays. (D) Binding interaction between miR-2053 and lncRNA *FOXD1-AS1 via* bioinformatics analysis. (E) Expression of miR-2053 *via* RT-qPCR analysis. (F) PC cell lines detection by Luciferase reporter assays. Mean ± SDs were calculated from N=3 independent and separate analyses with * *p* ≤ 0.05; NS *p* ≥0.05.
